# Estimation of Kidney Volumes in Autosomal Dominant Polycystic Kidney Disease: A Comparison Between Manual Segmentation and Ellipsoid Formula

**DOI:** 10.3390/clinpract15110191

**Published:** 2025-10-23

**Authors:** Nicola Maggialetti, Claudia Dipalma, Eva Colucci, Ilaria Villanova, Giovanni Lorusso, Maria Grazia Arcidiacono, Giovanni Piscopo, Amato Antonio Stabile Ianora

**Affiliations:** 1 Interdisciplinary Department of Medicine, Section of Radiology and Radiation Oncology, University of Bari “Aldo Moro”, 70124 Bari, Italy; 2 Nephrology and Dialysis Unit, “A. Perrino” Hospital, 72100 Brindisi, Italy; 3 Renal, Dialysis and Transplantation Unit, Department of Emergency and Organ Transplantation, University of Bari “Aldo Moro”, 70124 Bari, Italy

**Keywords:** autosomal dominant polycystic kidney disease (ADPKD), single kidney volume (SKV), computed tomography (CT), manual segmentation, ellipsoid formula

## Abstract

**Objectives**: Evaluate the agreement and interobserver variability between manual segmentation and the ellipsoid formula in estimating single kidney volume (SKV) in patients with autosomal dominant polycystic kidney disease (ADPKD). **Methods**: In this retrospective study, 130 unenhanced CT scans of ADPKD kidneys were analyzed. Three radiologists (one senior, two juniors) measured SKV using manual segmentation and the ellipsoid formula. Statistical analyses included intraclass correlation coefficient (ICC), Wilcoxon signed-rank test, Bland–Altman analysis, and paired *t*-tests to compare measurement values and computation times. **Results**: Both methods showed excellent interobserver agreement (ICC ≥ 0.977). No significant difference was observed in volume estimates between the two techniques (Wilcoxon *p* = 0.295). Bland–Altman analysis confirmed strong agreement between methods for the senior radiologist. The ellipsoid method was significantly faster for all readers (*p* < 0.05). **Conclusions**: The ellipsoid formula is a reliable, time-efficient alternative to manual segmentation for SKV estimation in ADPKD, offering comparable accuracy with reduced resource demands in clinical settings.

## 1. Introduction

Autosomal dominant polycystic kidney disease (ADPKD) is the most common inherited renal disease, with an estimated prevalence ranging from one in 1000 to one in 2500 individuals [1]. The disease is characterized by the gradual formation of large, fluid-filled cysts in the kidneys over several decades. These cysts cause significant kidney enlargement and, more critically, severely impair the functional integrity of the remaining healthy tissue [2,3]. ADPKD, therefore, is the fourth cause of end-stage renal disease (ESRD), representing about 10–15% of patients on dialysis and 10% of cases requiring renal replacement therapy [4]. ADPKD is classified into two types based on the mutated gene: type 1 ADPKD, caused by mutations in the PKD1 gene located on chromosome 16 (accounting for about 85% of cases), and type 2 ADPKD, caused by mutations in the PKD2 gene on chromosome 4 (present in about 15% of cases) [5,6]. Generally, mutations in PKD2 are associated with a later onset and a slower progression to renal failure compared to those in PKD1 [7,8].

Dysfunctional PKD1 or PKD2 genes result in non-functional proteins crucial for the normal organization of kidney tubules. This protein deficiency disrupts cellular processes, leading to the formation of renal cysts. This structural damage progressively impairs kidney function, eventually causing ESRD. Approximately half of ADPKD patients develop ESRD, typically around age 50, though the timing varies considerably among individuals [9].

Many individuals with ADPKD remain asymptomatic, with only a small proportion presenting early onset symptoms such as hypertension, abdominal pain, hematuria, urinary tract infections and nephrolithiasis [10]. Furthermore, ADPKD causes hepatic cysts in around 70% of patients and pancreatic cysts in about 5%, with cysts potentially affecting other organs like spleen, prostate, and seminal vesicles [11]. It also increases the risk of heart valve abnormalities, aortic aneurysms and intracranial aneurysms [12,13].

Conventional markers of kidney function, such as serum creatinine, estimated glomerular filtration rate (eGFR), and creatinine clearance, are not particularly effective in assessing the severity or progression of ADPKD. These indicators usually remain within a normal range or close to it until the disease reaches its later stages [14].

The Consortium for Radiologic Imaging Studies of Polycystic Kidney Disease (CRISP) demonstrated that total kidney volume (TKV) can track disease progression even before renal function is affected. Imaging plays a central role both in the diagnosis and the monitoring of the disease, and in the evaluation of treatment response. It is also useful in identifying and managing complications in ADPKD patients. Furthermore, TKV, assessed through magnetic resonance imaging (MRI), computed tomography (CT) or ultrasound (US), reflects the impact of enlarging renal cysts on the kidneys [12]. It is a clinically significant surrogate marker in ADPKD as it correlates with kidney function and serves as a predictor of functional disease progression, risk of ESRD and effectiveness of pharmacological treatment in slowing the disease’s progression [15,16].

There are various methods for measuring single kidney volumes (SKV), each differing in terms of complexity, accuracy, precision and time requirements. In manual segmentation, a radiologist delineates the renal contours on each image slice, including the parenchyma and all cysts, while excluding the renal pelvis and hilar structures. In the ellipsoid method, volume is estimated by measuring the kidney length in both coronal and sagittal planes, width at the maximum transverse diameter, and depth perpendicular to width, applying the standard ellipsoid formula [17].

The aim of this study is to compare the variability and concordance between manual segmentation and ellipsoid formula methods for measuring kidney volumes in patients with ADPKD.

## 2. Materials and Methods

### 2.1. Study Population

The study is a retrospective sub-analysis based on data prospectively collected within the ongoing multicenter pilot trial METROPOLIS, registered in the European Clinical Trials Database (EudraCT No. 2018-000477-77), which investigates the efficacy of Metformin and Tolvaptan in slowing disease progression of ADPKD. Preliminary results included data from the following participating centers: Giovanni XXIII Pediatric Hospital, Policlinico of Bari; Sant’Orsola-Malpighi University Hospital, Bologna; Spedali Civili University Hospital, Brescia; San Bortolo Hospital, Vicenza; University Hospital Ospedali Riuniti, Foggia; and IRCCS Casa Sollievo della Sofferenza, San Giovanni Rotondo. We opted to focus on SKV rather than TKV to more accurately estimate kidney size and avoid the averaging effect of measuring both kidneys together.

A total of 130 kidneys were analyzed and SKV was specifically calculated for each kidney. Inclusion criteria for patient selection were: (a) patients aged 18 or older; (b) confirmed genetic diagnosis of Type 1 ADPKD; (c) availability of CT imaging for renal volume assessment. Exclusion criteria were: (a) poor quality CT scan that does not allow adequate SKV evaluation; (b) patients affected from urinary tract obstruction; (c) patients with atypical (Type 2) or non-ADPKD cystic kidney disease.

### 2.2. CT Technique

CT scans were conducted at multiple centers, following standardized acquisition protocols with the required parameters: a single breath-hold scan spanning from the diaphragm to the pubic symphysis, utilizing a slice thickness of 0.625 mm. No intravenous iodinated contrast agent was used.

### 2.3. Manual Segmentation

**Figure 1:**
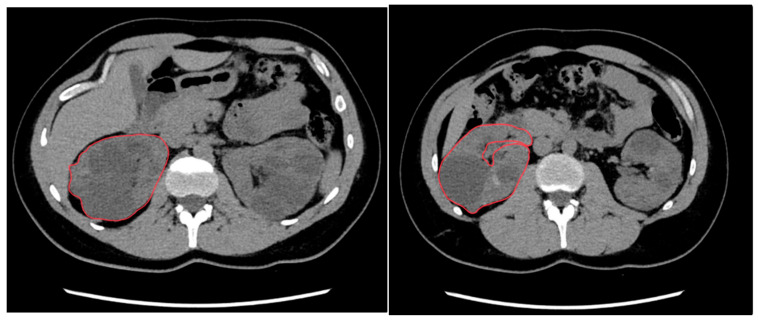
Renal volume calculation using manual segmentation. CT slices from a 45-year-old male patient with autosomal dominant polycystic kidney disease (ADPKD). The operator outlines the kidney contour (in red), excluding the hilar structures.

**Figure 2:**
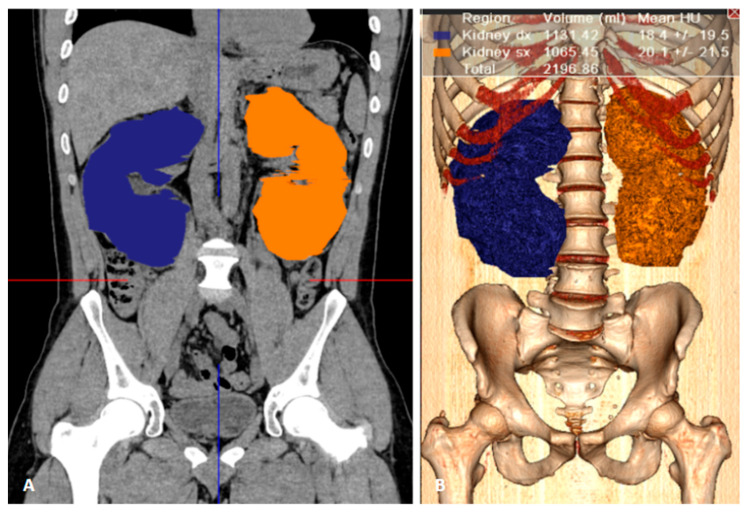
Renal volume calculation using manual segmentation. (**A**) Coronal plane showing the segmented area of both kidneys: right kidney (blue), left kidney (orange). (**B**) 3D reconstruction of the segmented renal volumes using reconstruction software (Vitrea FX 2.1, Vital Images, Minneapolis, MN, USA). Right kidney: 1131.42 mL; left kidney: 1065.45 mL. Total kidney volume (TKV): 2196.86 mL.

CT data were centrally transferred and analyzed on a workstation equipped with specialized image reconstruction software (Vitrea FX 2.1, Vital Images, Minneapolis, MN, USA). For each patient, multi-planar reconstructions (MPR) were generated in the axial, coronal, and sagittal planes to assess the renal parenchyma. Three operators with different experience in abdominal CT imaging (N.M. 10 years, C.D. 2 years, E.C. 2 years) independently performed slice-by-slice manual segmentation of both kidneys; delineation included the entire renal parenchyma and all cysts (exophytic cysts included), while excluding the renal pelvis and hilar vascular structures (Figure 1). Kidney volumes were calculated by the software after extrapolation of the surfaces and manual correction (Figure 2). The time taken by each reader to perform manual segmentation was manually recorded using a stopwatch.

### 2.4. Ellipsoid Formula

**Figure 3:**
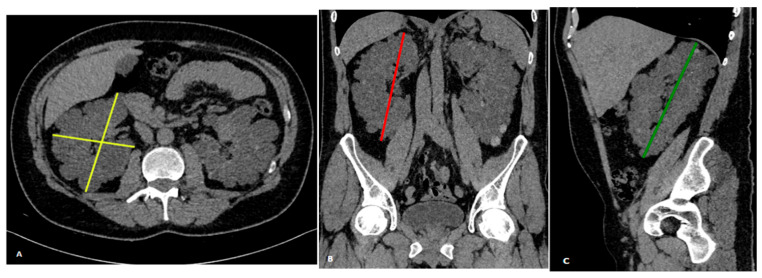
Renal volume calculation using the ellipsoid formula. 50-year-old male patient with autosomal dominant polycystic kidney disease (ADPKD). The operator measures the maximum kidney diameters in different planes using multi-planar reconstructions (MPR): (**A**) maximum axial diameters (yellow), (**B**) maximum coronal diameter (red), and (**C**) maximum sagittal diameter (green).

SKV was also estimated using the ellipsoid method, as proposed by Irazabal et al. [18]. For each kidney, the length was calculated as the average of the maximum longitudinal diameters measured in the coronal and sagittal planes. The width was taken from the transverse image at the maximum transverse diameter, while the depth was measured perpendicularly to the width in the same image (Figure 3). SKV was calculated independently by the three operators using the following formula: π/6 × [(coronal length + sagittal length)/2] × width × depth/1000.

All measurements were recorded in millimeters, and the time taken for the ellipsoid method was also manually recorded using a stopwatch.

### 2.5. Statical Analysis

SKV values obtained via manual segmentation and the ellipsoid formula were compared to assess agreement and accuracy between methods. Statistical analyses were performed using IBM SPSS Statistics, Version 30.0 (IBM Corp., Armonk, NY, USA). Interobserver agreement was evaluated by three raters with different years of experience (N.M. 10, C.D. 2, E.C. 2), who independently analyzed the same subset of 130 kidneys using both methods. It was evaluated using the intraclass correlation coefficient (ICC), calculated with a two-way mixed-effects model. Statistical comparisons between the two measurement approaches as well as among the values obtained by the three independent operators, were conducted using the Wilcoxon signed-rank test. Agreement between manual segmentation and the ellipsoid formula was further analyzed using Bland–Altman plots, with measurements from the senior radiologist (N.M.) considered the reference standard. We used a paired *t*-test to compare the computation times between two methods for each operator.

## 3. Results

Descriptive analysis of the 130 kidneys revealed that manual segmentation yielded SKV values ranging from 144.4 mL to 2293.0 mL (mean ± SD: 727.6 ± 454.5 mL). The ellipsoid method produced volumes ranging from 127.2 mL to 2458.6 mL (mean ± SD: 736.1 ± 494.6 mL). Interobserver agreement among the three readers was excellent for both techniques. For manual segmentation, the ICC was 0.998 (95% CI: 0.996–0.999). For the ellipsoid method, the ICC was 0.977 (95% CI: 0.952–0.990).

**Figure 4:**
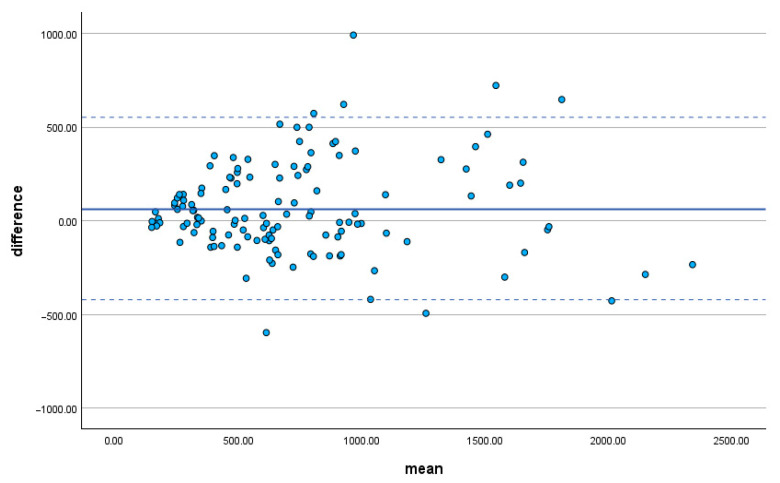
Bland–Altman plot illustrating the agreement between the ellipsoid method and manual segmentation for single kidney volume estimation. The solid line represents the mean difference between the two methods (65.78 mL), while the dashed lines indicate the 95% limits of agreement (±1.96 SD), with the upper and lower limits at +552.18 mL and −420.63 mL, respectively.

The comparison between the two methods using the Wilcoxon signed-rank test revealed no statistically significant difference in volume estimates (*p* = 0.295). Similarly, intra-rater comparisons showed no significant differences: for the senior radiologist (N.M.), the *p*-value was 0.295; for the junior radiologists (C.D. and E.C.), the *p*-values were 1.000 and 0.190, respectively. The agreement between manual segmentation and the ellipsoid formula was further illustrated by the Bland–Altman analysis, which was conducted using the measurements obtained by the senior radiologist (Figure 4).

The paired *t*-test, used to evaluate the computation times, showed a statistically significant difference between the ellipsoid method and manual segmentation for all three operators, with the ellipsoid method requiring less time and *p*-values of 0.00124 (N.M.), 0.00185 (C.D.), and 0.00155 (E.C.), all below the significance threshold of 0.05.

## 4. Discussion

ADPKD is a leading cause of ESRD and imposes a high socioeconomic burden due to frequent hospitalizations, lifelong antihypertensive treatments, progressive complications, and the eventual need for costly renal replacement therapy [19]. The progressive enlargement of renal cysts in ADPKD adversely affects patients’ quality of life, as it can lead to chronic flank or abdominal pain, intra-cystic hemorrhage, cyst infections, arterial hypertension, and nephrolithiasis, thereby contributing to increased morbidity and complicating clinical management [1,20]. Each cyst in a polycystic kidney behaves independently, but they are known to grow at a consistent rate. The cumulative growth of all these cysts in both kidneys results in an exponential increase in TKV, with larger and older cysts having a more significant impact on TKV changes than smaller, younger ones [21]. To identify potential treatments that may slow or halt the progression of ADPKD, it is crucial to identify effective biomarkers and evaluate their responses to novel therapies. A wide spectrum of biomarkers has been identified to assess the progression of ADPKD, including genetic, clinical, molecular, and imaging parameters. Genetic factors, particularly pathogenic variants in PKD1 and PKD2, have been strongly associated with disease severity, with truncating PKD1 mutations leading to earlier onset of kidney failure [22]. Molecular biomarkers, both serum-based (e.g., copeptin, FGF23, apelin) and urinary (e.g., KIM-1, MCP-1, microRNAs, and metabolomic profiles), provide insights into systemic and renal pathophysiology but remain largely investigational in clinical practice [23]. Clinical indicators such as age-adjusted eGFR, body mass index (BMI), male sex, and comorbidities (e.g., hypertension, cardiovascular disease) have shown prognostic value, with tools like the predicting renal outcome in polycystic kidney disease (PROPKD) score supporting individualized risk stratification [24]. Among all biomarker categories, imaging biomarkers have emerged as the most reliable and clinically validated tools to predict ADPKD progression. TKV, particularly when indexed in height-adjusted total kidney volume (htTKV), is the only qualified prognostic biomarker for ADPKD [25,26]. Data from the CRISP study demonstrated that baseline htTKV predicts future decline in kidney function: each 100 mL/m increase in htTKV raises the risk of developing chronic kidney disease stage 3 by approximately 1.48-fold, with changes in volume preceding measurable declines in eGFR by several years [12]. The Mayo Imaging Classification (MIC) further refines prognostication by adjusting htTKV for age and stratifying patients (aged ≥15 years) into five subclasses (1A–1E), where subclasses 1C–1E represent rapid disease progression [18].

TKV has been recognized as a significant imaging biomarker for assessing disease severity and predicting progression in ADPKD. The importance of TKV has been further reinforced by interventional studies. In the landmark TEMPO 3:4 trial, tolvaptan significantly slowed the increase in TKV and reduced the risk of clinical progression compared with placebo [27]. Subsequent trials such as REPRISE and TAME-PKD also confirmed the role of TKV as a key secondary endpoint [28,29]. Today, both the U.S. Food and Drug Administration (FDA) and the European Medicines Agency (EMA) recognize TKV as a qualified prognostic biomarker in ADPKD.

TKV measurement in ADPKD is commonly performed using manual, semi-automated, or fully automated segmentation methods based on imaging data. Manual segmentation remains the most frequently employed technique in research studies [30]. This approach involves the precise delineation of the kidney contours on every contiguous image slice, either by tracing or placing discrete points along the boundary. Although this method provides high accuracy and reproducibility, with error rates reported to be below 1.3% for kidney volume estimation [31], it is highly time-consuming, often requiring 15 to 30 min per kidney depending on image resolution and operator experience [32]. Manual segmentation also depends heavily on the skill and consistency of the operator, introducing potential inter- and intraobserver variability. Semi-automated segmentation methods have been developed to address these limitations by combining initial manual input with algorithmic processing. These approaches typically require the operator to provide seed points or initial contours, after which algorithms such as thresholding, region growing, watershed transformations, active contours, or graph-cut methods iteratively refine the segmentation [33,34]. Semi-automated techniques reduce segmentation time considerably and improve reproducibility by minimizing manual delineation, while maintaining accuracy comparable to fully manual methods. Nevertheless, these approaches can be sensitive to image quality issues such as noise, low contrast-to-noise ratio, and spatial heterogeneity, sometimes necessitating user correction or parameter adjustment during post-processing. While fully automated segmentation methods based on artificial intelligence (AI) are emerging, they often build upon semi-automated frameworks by incorporating shape models or machine learning algorithms. However, manual segmentation continues to serve as the reference standard for validating new methods [31]. Importantly, the choice between manual and semi-automated methods involves a trade-off between accuracy, processing time, and operator dependency. Semi-automated methods offer an efficient and reproducible alternative but require careful validation and standardization across datasets before routine clinical adoption. To facilitate this, there is a growing need for publicly available, annotated imaging databases and synthetic phantoms that enable rigorous benchmarking of segmentation algorithms [35]. Compared to segmentation, the ellipsoid method provides a faster and more streamlined approach for estimating both TKV and SKV. This technique relies on three linear measurements—length, width, and depth—combined in a geometric formula to approximate kidney volume. Specifically, SKV was calculated following the method proposed by Irazabal et al., where length is determined as the average of the maximum longitudinal diameters obtained from the coronal and sagittal planes, width is measured on the transverse image at the point of maximum transverse diameter, and depth is measured perpendicularly to the width on the same image [18]. In a study by Shi et al., ellipsoid-derived TKV demonstrated low bias and high precision when compared to manual segmentation [36].

We compared two different approaches for estimating SKV: the manual segmentation technique and the ellipsoid method. The primary aim was to assess the level of agreement between these methods and evaluate their interobserver variability across radiologists with varying levels of experience. In this study, we employed a manual volumetric segmentation approach, which involves manual delineation of the kidney contour on selected CT slices, followed by automatic interpolation across intermediate slices and manual correction of any that were not properly delineated by the software. The kidney volume was then computed using dedicated post-processing software.

The descriptive analysis revealed that the ellipsoid formula and manual segmentation methods yielded comparable volume estimates, with mean values of 736.1 ± 494.6 mL and 727.6 ± 454.5 mL, respectively. The range of values was similarly wide for both techniques, reflecting the substantial heterogeneity in kidney size within our sample. Notably, the Wilcoxon signed-rank test showed no statistically significant difference between the two methods (*p* = 0.295), suggesting that the ellipsoid approach can provide a reasonable approximation of renal volume when compared to the more detailed, but labor-intensive, manual segmentation. Interobserver agreement was excellent for both methods, particularly for the manual volumetric technique (ICC = 0.998). The ellipsoid method also demonstrated high interobserver reliability (ICC = 0.977), supporting its consistency even among radiologists with different levels of expertise. The analysis of the Bland–Altman plots highlights a satisfactory overall agreement between the two methods, with manual segmentation showing greater agreement in the assessment of larger volumes. Overall, the results of this study suggest that both the manual segmentation method and the ellipsoid formula method are reliable and accurate for estimating kidney volume. Accurate and reproducible TKV measurements, using an ultra-low-dose CT protocol and volume measurement are comparable to the reference standard of MRI planimetry [37].

Therefore, ultra-low dose CT protocol represents a viable alternative where the access to MRI is limited. Despite these encouraging results, some important considerations remain. The manual volumetric method, while more accurate due to its slice-by-slice assessment and volume reconstruction, is time-consuming and requires dedicated post-processing software, limiting its routine use in clinical settings. Nonetheless, the high level of agreement between the ellipsoid and manual volumetric methods, along with the demonstrated reproducibility of the ellipsoid technique, supports its potential role as a practical alternative in clinical scenarios where rapid volume estimation is required.

As reported by several authors [15,17,38,39,40] and confirmed by our own experience, the manual segmentation method is considerably more time-consuming. The results of the paired *t*-test showed a statistically significant difference in computation times between the two methods (*p*-values < 0.05), with the ellipsoid method being consistently faster than the manual segmentation method. The manual method required significantly more time, with senior operators taking around 15–20 min per kidney and junior operators up to 25–30 min. In contrast, the ellipsoid method reduced the time to approximately 3–5 min per kidney for senior operators and 5–6 min for junior operators. These findings suggest that the ellipsoid method is a more efficient approach for kidney volume assessment, offering a potential advantage in clinical and research settings where time efficiency is crucial. Driven by the need for simpler, less time-consuming, yet sufficiently accurate alternatives, researchers have increasingly focused on evaluating various methods for estimating SKV—including the ellipsoid method—and comparing their performance against manual segmentation technique. For instance, a study by Shi et al. [36] comparing the ellipsoid method with manual segmentation in patients with polycystic kidney disease reported similar levels of interobserver agreement (ICC = 0.991), supporting the reliability of the ellipsoid method for volume estimation.

Di Pietro et al. suggest that the performance of the ellipsoid method strongly depends on the operator’s experience, and that it is a useful method for estimating TKV with acceptable reliability only when carried out by an adequately experienced operator [41]. Nonetheless, despite these practical limitations, manual segmentation remains the reference standard for the assessment of SKV and TKV [17,38,39,40,42].

Kidney length has been proposed as a surrogate marker for disease progression in place of TKV measurement, given its ease of acquisition by ultrasound [43]. Although kidney length and volume, as assessed by MRI or CT, are linearly correlated, the precision of this correlation is low, limiting the utility of kidney length to a rough approximation of TKV. Moreover, Sharma et al. [38] indicates that kidney length lacks the sensitivity required to detect treatment-related changes and therefore should not be recommended as an outcome measure in clinical trials.

To overcome the time demands and operator dependency of manual methods, fully automated AI techniques are desirable. Recent studies have introduced novel approaches for automated kidney volume measurement [38,44,45,46,47,48,49], yet these methods are highly sensitive to image quality and prone to frequent failure, particularly in cases involving anatomically complex or highly cystic kidneys [50]. Furthermore, automated segmentations often exhibit high variability and substantial discrepancies compared to manual contouring [44]. As such, current AI methods are not sufficiently reliable to replace manual approach for detecting subtle longitudinal changes in kidney volume, as required in clinical trial settings. Nonetheless, deep learning–based segmentation offers additional advantages beyond efficiency and scalability. In particular, these methods have the potential to subsegment kidney structures, distinguishing cysts from renal parenchyma and enabling the extraction of novel imaging biomarkers. For example, exophytic cysts, those extending beyond the renal contour, can lead to overestimation of disease severity when included in TKV measurements. To address this, Bae et al. proposed identifying and excluding exophytic cysts, defined as those protruding more than 50% beyond the projected kidney boundary [51]. In response, Gregory et al. developed a deep learning approach capable of automatically detecting and quantifying cyst number, size, and parenchymal surface area, improving the prediction of eGFR decline and disease progression in ADPKD patients [52]. Moreover, automated identification of complex cysts—such as hemorrhagic or proteinaceous cysts that appear hyperintense on T1-weighted MRI—has also been shown to enhance prognostic accuracy, given their association with faster disease progression [53]. These advancements suggest that, while current automated methods may not yet be fully suited for precise TKV measurement in clinical trials, the integration of deep learning–based cyst characterization and parenchymal analysis holds promise for developing more refined, predictive imaging biomarkers in ADPKD [54,55].

Our study indeed has some limitations. Manual corrections during manual segmentation could introduce biases if inconsistent. The ellipsoid method, while simple, assumes kidney symmetry, which may not be accurate in cases with complex renal structures or pathology.

Similarly, manual segmentation depends on image quality and software performance, and errors in contour detection could affect accuracy despite manual corrections. Furthermore, this study was conducted across multiple centers, and despite the provision of standardized CT acquisition guidelines, inherent variability between scanners and imaging protocols at different sites could not be fully eliminated.

## 5. Conclusions

In conclusion, the ellipsoid method offers a reliable, efficient alternative to the more complex manual segmentation technique for estimating SKV, with comparable volume estimates and strong reproducibility. The manual method, although considered the gold standard for calculating renal volume, is more time-consuming and requires specialized tools making it less practical for routine clinical use. Both methods have their strengths, with the ellipsoid method serving as a practical choice for rapid volume estimation in clinical settings.

## Data Availability

The data presented in this study are available on request from the corresponding author due to institutional privacy restrictions.

## Abbreviations

The following abbreviations are used in this manuscript:

ADPKD	Autosomal Dominant Polycystic Kidney Disease
PDK1	Polycystic Kidney Disease 1
PDK2	Polycystic Kidney Disease 2
ESRD	End-stage Renal Disease
eGFR	Estimated Glomerular Filtration Rate
CRISP	Consortium for Radiologic Imaging Studies of Polycystic Kidney Disease
TKV	Total Kidney Volume
SKV	Single Kidney Volume
MRI	Magnetic Resonance Imaging
CT	Computed Tomography
US	Ultrasound
MPR	Multi-planar Reconstructions
BMI	Body Mass Index
PROPKD	Predicting renal outcome in polycystic kidney disease score
htTKV	Height-adjusted Total Kidney Volume
MIC	Mayo Imaging Classification
FDA	Food and Drug Administration
EMA	European Medicines Agency
ICC	Intraclass Correlation Coefficient
CI	Confidence Interval
